# Validity of the Khamis-Roche method, relative to bone age, in Portuguese children and adolescents from 11 to 15 years

**DOI:** 10.1016/j.jped.2026.101578

**Published:** 2026-07-03

**Authors:** Isabel Fragoso, Joao Albuquerque, Júlia Teles, Fabiana Bonito, Luís Miguel Massuça, Fernando Paiva Moura, Carlos Barrigas, Luis Alberto Flores

**Affiliations:** a Universidade de Lisboa, Faculdade de Motricidade Humana, Laboratory of Physiology and Biochemistry of Exercise, CIPER, Lisboa, Portugal; b Universidade do Porto, Faculdade de Medicina, Departamento de Biomedicina, Unidade de Bioquímica, Porto, Portugal; c Universidade de Lisboa, Faculdade de Ciências, Centro de Estatística e Aplicações, Lisboa, Portugal; d Universidade Lusófona de Humanidades e Tecnologias, Faculdade de Educação Física e Desporto, CIEFDES, Lisboa, Portugal; e Instituto Superior de Ciências Policiais e Segurança Interna, ICPOL, Lisboa, Portugal; f Autonomous University of Chihuahua, Faculty of Physical Culture Sciences, Chihuahua, Mexico

**Keywords:** Percentage of adult stature, Biological maturation, Somatic maturity, TW3, Anthropometry

## Abstract

**Objective:**

to validate maturity status classification, based on %PAH, relative to SA, in a sample of Portuguese children and adolescents of both sexes.

**Methods:**

BA was measured by the Tanner-Whitehouse-3 method, the PAH and %PAH were estimated by the Tanner-Whitehouse-3 method (BA- PAH, % BA-PAH) and the Khamis-Roche method (KR- PAH, %KR- PAH) in 371 children (171 girls). Intraclass Correlation Coefficients and Bland-Altman plots were performance between BA-PAH / BA-%PAH and KR- PAH/%KR- PAH methods. Two-way Analysis of Variance (ANOVA) was made to evaluate the effect of sex and maturity status.

**Results:**

The average mean differences between BA-PAH and KR-PAH was 0.2 ± 4.2 cm in boys and 0.6 ± 3.1 cm in girls (p > 0.05) and between BA-% PAH and KR-% PAH was -0.1 ± 2.1% in boys and 0.4 ± 1.8% in girls (p > 0.05). A high interclass correlation coefficient was higher than 0.91 for BA-% PAH and KR-% PAH. Early maturity status was related to negative differences between BA- KR PAH.

**Conclusion:**

KR % PAH presented a good agreement and might be a valid estimate of biological maturity status in average maturing children and adolescents.

## Introduction

Biological maturation is considered an important factor in paediatric research, since it is known to relate significantly with growth, auxological changes during puberty, cognitive, social-affective and motor performance [1]. Maturity estimates are often used in a clinical setting, in order to diagnose unusual growth, identify the need for therapeutic intervention, and provide family counselling [2]. Additionally, in youth sports talent identification and selection models, matching children according to their maturational status is seen as an issue of major significance, in order to equalize competition, [3] enhance the chance for success, [4,5] and reduce injury rates [6]. Maturity assessment is especially important during puberty, since biological variability between individuals of the same chronological age (CA) is greater during this period [7].

Bone age (BA) is considered the gold standard of biological maturity indicators [[8], [9], [10]]. It has proven to be an accurate estimator, does not require the use of longitudinal data, is applicable to both sexes, and throughout the entire growth process [1,11]. This method presents, however, several limitations, since it requires the interpretation of highly trained examiners and use of specialized equipment, is costly, time-consuming, and incurs safety and ethical issues involving the use of a low dose of radiation, for a hand-wrist radiograph [12]. The percentage of adult height, achieved at any point during the growth period, can also be used as an indicator of maturity status [13,14]. Although a precise measure of adult height requires the use of longitudinal data, [10] predictive equations have been proposed, based on SA, but also on other, non-invasive alternatives [15,16]. Non-invasive maturity indicators are particularly advantageous in a non-clinical setup, such as a sports organization, since it would be a safe, inexpensive, quick and simple way of assessing large samples of young athletes. Given that measures of bone dimension are essentially genetically determined, parental height has traditionally been used as a surrogate proxy of a child's genetic growth potential at a population level [17]. However, when assessing the growth or predicting the adult stature of an individual child, simple mid-parental target height is an unreliable and imprecise estimate due to polygenic variation, regression to the mean, and environmental factors such as prenatal development, nutrition, and lifestyle [1,18]. To overcome the poor individual predictive accuracy of isolated parental metrics, multivariate regression models, such as the Khamis-Roche method, are required to significantly reduce prediction errors by contextualizing the unadjusted midparental stature alongside the child’s own phenotypic data [7]. Khamis-Roche method, is one of the most commonly used non-invasive approaches for estimating adult height, which uses the children's CA, current height, body mass, and midparental height [15]. The percentage of predicted adult height has been previously used in studies of physical activity and youth [12,19,20]. However, the evidence is limited concerning the validity of this indicator. Recently, Parr et al., [21] observed a higher capacity of prediction of Peak Heigh Velocity (PHV) by the Khamis-Roche method in comparison with other non-invasive methods, in longitudinal data of young soccer players. In addition, prior research has found only moderate agreement between maturity status classified by BA and by the percentage of predicted adult height (%PAH), in samples of youth athletes. A first cross-sectional study, conducted with 143 US male youth football players, 9–14 years of age, [22] and a more recent study, using a sample of 180 Portuguese male youth soccer players, aged 11–14 years, [23] found generally moderate agreement (52–69%), and low to moderate kappa coefficients (0.23–0.50) and Spearman rank-order correlations (0.27–0.55), between both maturity estimates. This led the authors to suggest that %PAH may have limitations when used to constitute homogeneous groups of youth according to maturity status, for example, in sportive talent development programmes. Given the importance of non-invasive maturity estimates, and the absence of reference data using a non-athletic sample of boys and girls, the purpose of the present study is to validate maturity status classification, based on %PAH, as accessed by the Khamis & Roche (KR) method, relative to SA, in a sample of Portuguese children and adolescents of both sexes.

## Methods

### Sample

The present analytic cross-sectional study was conducted among three state-funded school institutions in Portugal, which previously allowed for the intervention. The data collection period was defined between March and June 2012. From an initial sample of 459 participants who provided parental written consent, together with an initial biosocial questionnaire, 86 were excluded from the study, for not filling the questionnaire correctly, and 2 for not attending school on the day scheduled for assessment. A final sample of 371 participants of both sexes, 200 boys and 171 girls, between 11 and 15 years of age, was assessed. All participants were of Portuguese nationality, but not all were of Portuguese ancestry. In total, 10.0% of the boys (n = 20), and 12.9% of the girls (n = 22) had non-Portuguese ancestry (in boys: five boys had at least one of the parents of African ancestry from Angola, Cape Verde, Mozambique, Guinea-Bissau or S. Tomé e Principe, five had Brazilian ancestry, nine East European ancestry such as Moldavia, Romania, Ukraine or Russia and one other nationality. In girls: twelve participants with at least one of the parents from a Portuguese-speaking African country, seven of Brazilian ancestry, two of Eastern European ancestry, and four of other nationality. The authors have decided to include these participants in the study since, based on the most recent national survey, they believe this variability is representative of the current Portuguese population.

The presence of physical or mental disability was an exclusion criterion. The study was authorized by a local ethics committee and performed according to the Helsinki declaration.

### Measures

#### Biosocial information

A biosocial questionnaire (*RAPIL II)*, was used to gather personal, academic, family-related and socio-economic information, which included date of birth, ethnicity, parental self-reported height, nationality and age. Due to parents and household-related information, the questionnaire was handed out with instructions to fill in at home, and return in a sealed envelope. The questionnaire was handed out 2 weeks prior to the assessment at school, which was only performed to whom returned this document correctly filled out. It takes around 15–20 min to fill in.

#### Anthropometric measures

Body mass and standing height were obtained according to the ISAK protocol. Body mass was measured to the nearest 0.05 kg, using a Secca body scale, model 761 7019,009 (Vogel & Halke, Hamburg, DE). Height was measured to the nearest mm (0.1 cm), using a Siber-Hegner anthropometric kit (DKSH Ltd., Zurich, SW). All measures were taken by the same accredited ISAK anthropometric technician (level 2), in a private and heated room within school facilities. Participants wore shorts and a t-shirt, and shoes were removed. In a prior pilot study, using 20 participants of the same ages and constitution as the ones in the sample, the intra-observer technical error of measurement (TEM) was nonexistent for weight, and of 0.29 cm for height, well within the limit of 1%.

#### Bone age

Biological maturity was assessed through BA evaluation, according to the Tanner-Whitehouse III Method (TW3 method) [10]. The maturity ratings were performed by a trained examiner, blinded to the CA of the participants. A portable X-ray device, model Ascot 110, was installed in a private area and used to take left-hand wrist radiographs. This X-Ray device operates with a low level of radiation (set to 3 mA/s and 36 kV or 5 microsieverts), and is free from radiation leakage, as monitored through individual dosimetry. The equipment was licensed by the Directorate-General of Health (DGH), and its use was performed according to the legal requirements in force, and supervised by a radiologist. Kodak MIN-R 2 frameworks were used to place the X-ray films (Kodak medical X-ray film, 18 × 24 cm). All films were developed with a Gevamatic 60 film processing machine, using AGFA G-153 developer and G-354 fixer fluids. A set of 10% (n = 37) set of radiographs was assessed by an independent observer. Mean (± SD) difference between assessments was of 0.03 (±0.04) year, and inter-observer TEM was 0.12 years.

#### Maturity estimation

CA, standing height, body mass, and midparental height ([father’s height +mother’s height]/2) were used to predict adult height (PAH), according to the KR equation [15]. The KR method was developed using the Fels longitudinal sample, and presents an average median absolute deviation (MAD) 90% error bound of 5.34 cm for boys, and 4.25 cm for girls, between predicted and actual adult height. Parental self-reported height was adjusted for overestimation, according to the reference values specific for sex and age group in the NHANES survey [24]. Corrected mean values of parental height for the current sample were: 174.08 ± 6.95 cm for father’s height, 161.57 ± 6.52 cm for mother’s height, and 167.83 ± 5.20 cm for midparental height. Adult height was equally estimated, based on SA. In both methods, the current height of the subject was expressed as a %PAH, as an estimate of biological maturity status. Based on the difference between BA and CA (BA-CA), participants were classified into three maturity categories. If the subject was within the band of ±1 year of BA-CA, it was classified as on time, or average maturing; if BA was behind CA by >1 year, late maturing; and if BA was advanced relatively to CA by >1 year, early maturing. The band of ±1.0 years is similar to the standard deviations of BA within specific CA groups between 11 and 16 years [3].

### Statistical analysis

Descriptive statistics for the sample were calculated, using rounded decimal age to define age groups (eg., 11 years: decimal age between 10.50 and 11.49). Reliability analysis (Intraclass Correlation Coefficients and Bland-Altman plots) for PAH, and achieved %PAH, between BA and the KR method, was performed. Two-way Analysis of Variance (ANOVA) was made to evaluate the effect of sex and maturity status, on the differences between PAH and %PAH, as assessed by both methods. Statistical analysis was performed using the SPSS (Statistical Package for the Social Sciences Inc, Chicago, IL) software, version 21.0, considering a significance level of 5%.

## Results

Descriptive statistics for height, weight, BA, BA−CA, PAH and %PAH, based both on BA and the KR method, BA−KR PAH and BA−KR %PAH, for each age group, can be found in Table 1, Table 2, respectively, for the male and female samples. In the boys sample, BA–CA is negative from 11 to 13y, as well as considering the entire sample (BA−CA = −0.42 ± 1.41y), whereas girls on the other hand, seem to present relatively average maturational development, both in each age group, and considering the entire sample (BA−CA = 0.07 ± 1.06y). Range for BA−CA is however, similar in both sexes (−3.77 to 3.19y for boys; −3.62 to 3.19y for girls). Table 1, Table 2 present the descriptive characteristics of male and female participants, respectively.

**Table 1:** Descriptive statistics for height, weight, BA, BA−CA, PAH and %PAH, based on BA and the KR methods, BA-KR PAH and BA-KR %PAH, for the male sample, by age group (n = 200).

	11 years (n = 35)			12 years (n = 52)			13 years (n = 45)			14 years (n = 34)			15 years (n = 34)		
Variable	Mean ± sd	Min	Max	Mean ± sd	Min	Max	Mean ± sd	Min	Max	Mean ± sd	Min	Max	Mean ± sd	Min	Max
Weight (kg)	39.5 ± 8.3	26.0	60.5	43.7 ± 12.0	26.0	99.5	49.0 ± 10.9	27.0	78.0	55.0 ± 11.0	35.0	83.0	62.6 ± 11.7	45.0	94.0
Height (cm)	144.5 ± 7.6	132.4	164.5	150.1 ± 8.7	133.7	167.0	157.2 ± 9.3	132.3	179.9	165.3 ± 8.9	148.7	187.7	170.7 ± 7.0	159.7	191.8
BA (years)	10.08 ± 1.27	8.08	13.61	11.00 ± 1.51	8.01	13.77	12.68 ± 1.31	10.31	16.50	14.14 ± 1.37	11.68	16.50	15.36 ± 1.21	12.83	16.50
BA −CA (years)	−0.98 ± 1.20	−2.98	2.11	−0.97 ± 1.47	−3.77	1.71	−0.33 ± 1.29	−2.94	3.19	0.11 ± 1.44	−2.82	2.70	0.35 ± 1.12	−1.97	1.94
BA PAH (cm)	178.2 ± 6.0	167.6	190.7	178.4 ± 6.3	161.6	190.0	177.6 ± 6.5	159.0	192.9	177.2 ± 6.7	165.1	193.1	176.5 ± 7.7	161.5	204.9
KR PAH (cm)	176.8 ± 7.0	165.8	189.8	177.0 ± 7.1	161.1	192.6	177.6 ± 6.9	159.7	193.4	178.9 ± 7.4	165.4	196.1	177.0 ± 5.9	166.0	193.5
BA %PAH	81.0 ± 2.0	78.3	88.2	84.1 ± 2.8	80.0	91.0	88.5 ± 3.0	83.2	99.2	93.3 ± 3.6	88.1	99.2	96.7 ± 2.8	90.8	99.2
KR %PAH	81.7 ± 1.9	78.6	86.8	84.7 ± 2.6	80.0	93.6	88.5 ± 3.2	82.4	97.2	92.4 ± 2.3	87.1	97.6	96.4 ± 1.8	92.5	99.7
BA -KR PAH (cm)	1.5 ± 3.0	−5.3	7.6	1.4 ± 4.1	−8.7	9.6	<0.1 ± 3.9	− 11.5	6.6	−1.7 ± 4.8	−14.6	6.5	−0.5 ± 4.7	−6.6	11.4
BA -KR %PAH	−0.7 ± 1.4	−3.5	2.4	−0.6 ± 1.9	−4.7	4.1	<−0.1 ± 1.9	−3.6	5.3	0.9 ± 2.5	−3.3	7.4	0.3 ± 2.5	−5.5	3.6

**Table 2:** Descriptive statistics for height, weight, SA, SA−CA, PAH and %PAH, based on BA and the KR methods, BA-KR PAH and BA-KR %PAH, for the female sample, by age group (n = 171).

	11 years (n = 33)			12 years (n = 46)			13 years (n = 27)			14 years (n = 32)			15 years (n = 33)		
Variable	Mean ± sd	Min	Max	Mean ± sd	Min	Max	Mean ± sd	Min	Max	Mean ± sd	Min	Max	Mean ± sd	Min	Max
Weight (kg)	40.6 ± 9.2	27.0	58.0	45.4 ± 12.5	29.0	104.0	49.0 ± 9.5	38.5	72.0	51.8 ± 9.1	34.0	72.0	55.9 ± 10.0	41.0	81.0
Height (cm)	146.3 ± 8.2	133.0	161.5	151.7 ± 6.1	138.3	164.3	155.4 ± 5.7	146.2	165.1	158.6 ± 6.5	143.1	168.0	162.8 ± 7.1	148.6	183.9
BA (years)	11.32 ± 1.26	8.50	14.33	11.84 ± 1.32	8.42	15.00	13.11 ± 1.16	10.85	15.00	14.21 ± 1.01	11.99	15.00	14.93 ± 0.49	12.86	15.49
BA −CA (years)	0.27 ± 1.14	−2.61	3.19	−0.14 ± 1.30	−3.62	2.91	0.13 ± 1.15	−1.73	2.29	0.21 ± 0.95	−2.08	1.27	−0.01 ± 0.43	−1.77	0.49
BA PAH (cm)	163.3 ± 5.7	155.3	172.8	164.7 ± 5.0	150.9	178.2	162.6 ± 5.8	153.1	175.1	161.9 ± 6.7	144.6	176.1	164.5 ± 7.2	150.1	185.4
KR PAH (cm)	164.1 ± 4.7	155.4	172.5	164.7 ± 4.4	155.0	178.4	163.4 ± 4.5	155.4	171.1	163.0 ± 5.4	151.3	172.6	164.9 ± 6.9	150.1	185.6
BA %PAH	89.6 ± 3.2	83.6	97.7	92.1 ± 3.3	85.2	99.1	95.5 ± 2.5	90.1	99.1	98.0 ± 1.6	93.5	99.1	99.0 ± 0.5	96.8	99.8
KR %PAH	89.1 ± 3.4	83.2	96.3	92.1 ± 2.7	87.0	100.0	95.1 ± 1.9	91.5	98.7	97.3 ± 1.4	93.7	99.4	98.7 ± 0.8	96.3	100.0
BA -KR PAH (cm)	−0.9 ± 3.8	−8.6	8.0	<−0.1 ± 3.6	−8.1	9.4	−0.7 ± 3.5	−6.1	7.0	−1.1 ± 2.4	−7.9	4.5	−0.4 ± 1.2	−4.7	2.0
BA -KR %PAH	0.5 ± 2.1	−4.5	5.0	<0.1 ± 2.0	−5.4	5.0	0.5 ± 2.0	−3.9	3.6	0.7 ± 1.4	−2.6	5.1	0.3 ± 0.7	−1.1	2.8

**Fig. 1:**
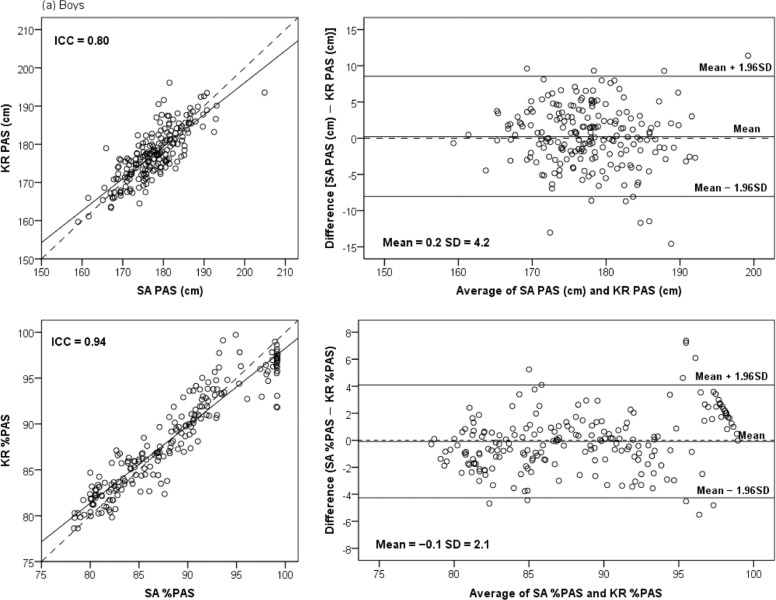
Scatter plot with concordance (dashed line) and regression (solid line) lines, and Bland-Altman plots, to evaluate agreement between BA PAH and KR PAH, and between BA %PAH and KR %PAH, in the boys sample.

**Fig. 2:**
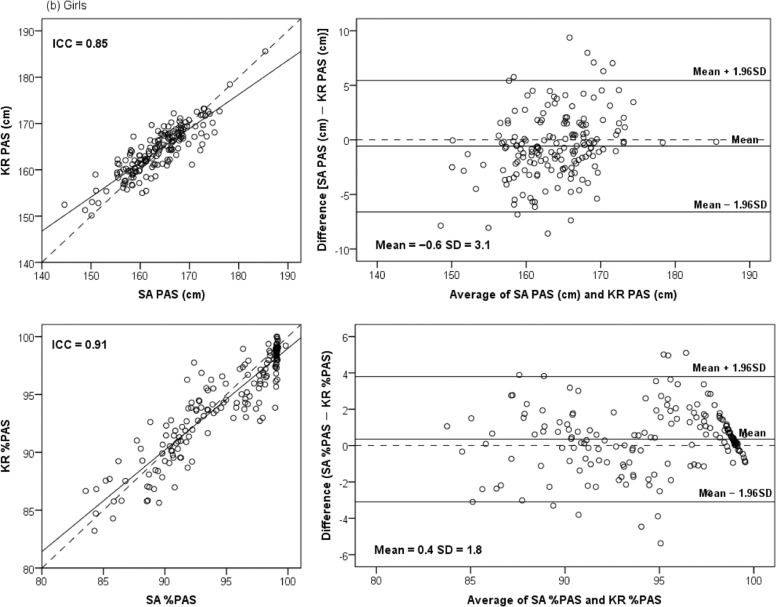
Scatter plot with concordance (dashed line) and regression (solid line) lines, and Bland-Altman plots, to evaluate agreement between BA PAH and KR PAH, and between BA %PAH and KR %PAH, in the girls sample.

Reliability analysis of PAH and %PAH assessed by the KR method, compared to SA, for both sexes, is presented in Table 3. Scatter plots with regression and concordance lines, and Bland-Altman plots are presented in Fig. 1, Fig. 2, for boys and girls, respectively. Concerning PAH, ICC values reveal a general strong agreement between BA and the KR method, of 0.80 for boys, and 0.85 for girls. ICC is higher for %PAH in both sexes, of 0.94 for boys, and 0.91 for girls. BA−KR PAH and BA−KR %PAH present a respective mean value of 0.2 ± 4.2 cm and −0.1 ± 2.1% for boys, and −0.6 ± 3.1 cm and 0.4 ± 1.8% for girls. Even though the mean difference for both assessment methods is low, BA−KR PAH ranges from −14.6 to 11.4 cm for boys, and −8.6 to 9.4 cm for girls, and BA−KR %PAH ranges from −5.5 to 7.4% for boys, and −5.4 to 5.1 for girls.

**Table 3:** Intraclass Correlation Coefficient (ICC) and Bland-Altman Limits of Agreement (LoA), for PAH and %PAH, assessed by BA and the KR method, for both sexes.

	Boys (n = 200)					
Variable	Intraclass Correlation Coefficient		Bland-Altman			
	ICC	95% CI	Mean diff ± s.d.	LoA	Min	Max
PAH	0.80	(0.75, 0.85)	0.2 ± 4.2	(−8.1, 8.6)	−14.6	14.4
%PAH	0.94	(0.92, 0.95)	−0.1 ± 2.1	(−4.3, 4.1)	−5.5	7.4

	Girls (n = 171)					
Variable	Intraclass Correlation Coefficient		Bland-Altman			
	ICC	95% CI	Mean diff (s.d.)	LoA	Min	Max
PAH	0.85	(0.80, 0.89)	−0.6 ± 3.1	(−6.6, 5.5)	−8.6	9.4
%PAH	0.91	(0.88, 0.94)	0.4 ± 1.8	(−3.1, 3.8)	−5.4	5.1

**Fig. 3:**
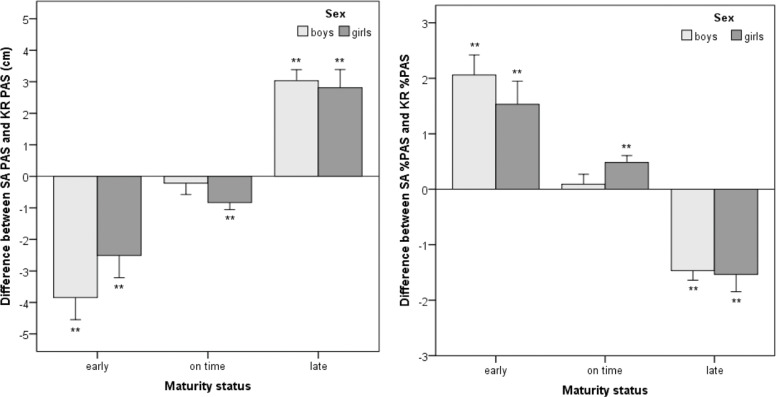
Difference (mean ± SE) between BA PAH and KR PAH (cm), and BA %PAH and KR %PAH, according to sex and maturity status. ** Mean value differs significantly from 0 (p < 0.001).

Descriptive statistics for the differences between BA and CA, BA PAH and KR PAH, and BA %PAH and KR %PAH, divided by sex and maturity status are presented in Table 4. Differences between BA and KR PAH and BA and KR %PAH are further represented in Figure 3. A moderate negative correlation level is observed between BA−CA and BA−KR PAH, for boys (*r* = −0.63; p < .001) and girls (*r* = −0.51, p < .001), and a moderate positive correlation of the same magnitude is shown between BA−CA and BA−KR %PAH. BA PAH−KR PAH and BA % PAH−KR %PAH differ significantly from 0 in early and late maturing boys (p < .001), but not in average maturing boys (p = 0.55 for PAH and p = 0.61 for %PAH). Considering all maturity groups together, neither BA−KR PAH (p = 0.41) nor BA−KR%PAH (p = 0.56) differs significantly from 0. BA PAH−KR PAH, and BA %PAH −KR %PAH differ significantly from 0 in early, average, and late maturing girls (p < 0.001), as well as considering all maturity groups together (p = 0.01). In the boys sample, a moderate negative correlation (*r* = −0.51, p < .001) was still found between midparental height and BA PAH−KR PAH, and a moderate positive correlation of equal value between midparental height and BA %PAH −KR %PAH. No significant association was found between midparental height and BA PAH−KR PAH (*r* = −0.07, p = .388), or BA %PAH −KR (*r* = 0.06, p = .477), in the girls' sample.

**Table 4:** Descriptive statistics for the difference between height assessment, using BA and the KR method, by maturity status, for both sexes.

Sex	Maturity status	N	BA-CA (years)			BA-KR PAH (cm)			BA-KR PAH (%)		
			Mean ± SD	Min	Max	Mean ± SD	Min	Max	Mean ± SD	Min	Max
Boys	Early	38	1.56 ± 0.53	1.00	3.19	−3.8 ± 4.3⁎⁎	−14.6	6.8	2.1 ± 2.2⁎⁎	−3.4	7.4
	On time	91	−0.05 ± 0.58	−0.99	0.99	−0.2 ± 3.4	−11.5	9.3	0.1 ± 1.7⁎⁎	−4.8	5.3
	Late	71	−1.95 ± 0.66	−3.77	−1.02	3.0 ± 3.0⁎⁎	−4.0	11.4	−1.5 ± 1.4⁎⁎	−5.5	1.9
Girls	Early	26	1.67 ± 0.61	1.03	3.19	−2.5 ± 3.6⁎⁎	−8.6	8.0	1.5 ± 2.1⁎⁎	−4.5	5.1
	On time	121	0.07 ± 0.50	−0.98	0.95	−0.8 ± 2.5⁎⁎	−7.4	9.4	0.5 ± 1.4⁎⁎	−5.4	3.9
	Late	24	−1.66 ± 0.70	−3.62	−1.01	2.8 ± 2.8⁎⁎	−2.8	7.1	−1.5 ± 1.5⁎⁎	−3.9	1.5

⁎⁎ Mean value differs significantly from 0 (p < 0.001). BA, Bone age.

Two-way Analysis of Variance (ANOVA) tests were performed to evaluate the effect of sex and maturity status, and their interaction, on SA−KR PAH and SA−KR % PAH. Normality and homogeneity of variances assumptions of two-way ANOVA were checked. Sex, and interaction between sex and maturity status did not show a significant effect either for SA−KR PAH (*F*(1365) = 0.178, p = 0.673; and *F* (2365) = 2.295, p = 0.102, respectively), or SA−KR %PAH (*F*(1365) = 0.111, p = 0.739; and (*F* (2365) = 1.992, p = 0.138, respectively). Significant main effect of maturity status was observed for both SA−KR PAH (*F*(2365) = 64.36, p < .001; partial eta-squared = 0.261, high effect) and SA−KR %PAH (*F*(2365) = 67.295, p < 0.001; partial eta-squared = 0.269, high effect). Post hoc analyses performed using the Tukey test revealed significant differences between all maturity groups for SA−KR PAH and SA−KR %PAH (p < .001).

## Discussion

The present study analyses the correspondence between the %PAH based on the Khamis-Roche relative to the BA method in Portuguese children and adolescents. The findings indicated a general strong to very strong agreement between BA PAH and KR PAH, and BA %PAH and KR %PAH in both sexes. Higher ICC values for %PAH than for PAH may be related to data heterogeneity and variability differences concerning both measures; [25] nevertheless, it is important to consider that the relative error inherent to each individual remains the same with both measures, since %PAH is estimated from PAH. In a previous study, the authors analysed the relation between BA %PAH and KR %PAH using a population sample of 6 to 16 years, observing an agreement between both methods in all age groups of both sexes based on the Bland-Altman technique [26]. That study was one of the first to compare the BA %PAH and KR %PAH in general populations; the majority in the literature were in soccer children and adolescent players.

Furthermore, in the present work, it was observed that when comparing groups of maturity status (early, average and late maturing participants), defined according to BA and the KR method, agreement between both methods seems to be drastically reduced. In a study conducted with 143 male youth football players (9.27 to 14.24 years of age), Malina et al., [22] found only moderate concordance levels between both methods of maturity classification, of 62% (53–69%), a kappa coefficient of 0.46 (0.19–0.59), and a Spearman rank-order correlation of 0.52 (p < 0.001). In a more recent study, using a cross-sectional sample of Portuguese male youth soccer players (11–14 years), Malina et al., [23] have found similar values between both methods: raw agreement between 57–63%, kappa coefficients of 0.23, and a Spearman rank-order correlation between 0.27–0.47 (p < 0.001). Flores et al., [27] observed similar results when validating a new method to predict BA based on the percentage of adult height by the Khamis-Roche method and using the BA observed by TW3 as a criterion method; the degree of agreement was moderate when classifying the sample by maturity status (late, average, and early) to compare both methodologies. In that same study, no differences were found between BA %PAH and KR %PAH when the whole sample was compared.

In relation to the present work, the authors should equally consider that, although the mean difference between both assessment methods is low, dispersion around the mean values is somewhat elevated. The total range of variability between SA−KR PAH and SA−KR %PAH, reflects even wider differences between both assessment methods, which may have a significant impact when dividing the sample into groups according to maturity rate, using different indicators.

These findings led the authors to suggest that non-invasive indicators may have limitations when used to divide samples according to maturity status. This limitation has also been observed in methodologies for estimating the peak height velocity (PHV), where the prediction of non-invasive methods (which commonly use chronological age and anthropometric measures as predictor variables) tend to approach the mean, resulting in a later prediction of PHV in early maturers, and in an earlier prediction of PHV in late maturers; [28] especially when working with small samples, as was observed in the Wrocław Growth study by Koziel and Malina [5] and for Malina et al., [29] in the Longitudinal Fels study.

Concerning the data from the present study, PAH and %PAH, estimated by the KR method, differ significantly from SA in early and late maturing boys, but not in average maturing boys. This difference is also not significant when considering all the boys' samples together. These results suggest that the KR method may be an accurate maturity estimator for average maturing boys, but not for early and late maturing boys. For this reason, it seems reasonable to suggest that this indicator should be used together with an independent maturity estimator, in order to adjust predictions to the child’s maturity status. Non-significant results when accounting for the entire boys sample may, on the other hand, be a consequence of similar error magnitude for early and late maturing boys. In fact, %PAH by the KR method seems to be underestimated in early maturers, and overestimated in late maturers, with approximately the same magnitude, and thus the inherent error in both groups may be annulled when analyzing the whole sample. This corroborates that it may be desirable to divide boys' samples according to maturity status in studies using the KR method to estimate PAH or %PAH.

In the girls' sample, on the other hand, the KR method seems to differ significantly from BA in early, average, and late maturing girls, as well as considering all maturity groups together, both for PAH and %PAH. Systematic error in the KR equation may explain the results for average maturing girls and the whole sample, whether maturity-related growth differences may explain the differences in early and late maturing girls, similarly to what happened in the boys sample.

In other findings, moderate negative correlation levels between BA−CA and BA−KR PAH for girls, and moderate positive correlation of the same magnitude between BA−CA and BA−KR %PAH, respectively for boys and girls, together with the ANOVA results, corroborate the suggestion that bone maturity significantly influences height prediction using the KR method. This means that KR PAH will be overestimated and KR %PAH underestimated in early maturing individuals, and the opposite will happen in late maturing subjects. This occurs because the KR method only accounts for the subject’s CA and not their maturation timing, and therefore will overestimate PAH and underestimate %PAH in a child who is closer to the end of their maturational process, with the opposite being true for a child who is farther from their mature state. Put into practice, the same 13-year-old child may have a BA of 14 years of age, or 12 years of age, and that will not influence prediction using the KR method, which only accounts for the average developed children. In the first case, the child is already closer to adult height than the KR prediction will estimate, and will be farther from achieving adult height in the second case. Similar results have been found in previous work [22].

Furthermore, mid-parental height showed a significant main effect concerning the influence of maturity in PAH and %PAH. In the boys sample, significant moderate correlation levels were also found between midparental height, and BA PAH−KR PAH, and BA %PAH −KR %PAH. This means that KR PAH seems to be overestimated, and KR %PAH underestimated, in boys with very tall parents, with the opposite happening for very short parents. In other words, when comparing midparental height to BA−KR PAH and BA−KR %PAH, differences are higher, and opposite, for taller and shorter parents, probably meaning that the relative importance given to midparental height in the KR equation is overestimated when parents’ height differs greatly from the population average. In the girls' sample, no significant relation was found between mid-parental height and BA PAH−KR PAH.

As main limitations of the present study, the authors consider that the use of self-reported parental height, even adjusted for overestimation, may influence the reliability of this measure. Directly measured parental height may therefore improve prediction accuracy, using the KR method. Based on the father’s height, compared with reference standard values for the Portuguese population, boys in the current sample may also be somewhat taller than the mean population height, even though maturity estimates should not be influenced by this fact. Additionally, the KR method was validated solely against the American population, [30] as the TW3 method was validated using different populations of European and American ancestry [10]. Finally, this study was limited to a cross-sectional sample, even that one of considerable dimension. Further insight may be provided when analysing the stability of prediction over a determined course of time, or when comparing predicted values with actual growth values, using a longitudinal sample.

In conclusion, the precision of KR PAH and KR %PAH is influenced by children’s SA, in both sexes. In boys, the KR method also seems to be influenced by a very tall or very short midparental height. KR %PAH may be a reasonably valid estimate of biological maturity status in average maturing boys. Using the KR equation may therefore be limited when used in samples that are typically early maturing, like male samples of youth athletes. The additional use of an independent indicator of maturity status may help to improve prediction accuracy in this case. The graphical abstract summarizing the main findings of this study is available as Supplementary Material.

## Data availability statement

The datasets analyzed in the present study are available from the corresponding author on reasonable request.

## Funding statement

This study was funded by the Fundação para a Ciência e Tecnologia (FCT) project [PTDC/DES/113156/2009].

## Permission to reproduce material from other sources

The authors confirm that this manuscript does not reproduce any figures, tables, or other material from previously published sources. All content included in the manuscript is original and created by the authors. Therefore, no permission to reproduce material from other sources is required.

## Conflicts of interest

The authors report no conflict of interest.

## Appendix Supplementary materials

Image, application 1
