# Supplementary figures and images for: Validity of the Khamis-Roche method, relative to bone age, in Portuguese children and adolescents from 11 to 15 years
Source: J Pediatr (Rio J). 2026 Jul 3;102(5):101578. doi: 10.1016/j.jped.2026.101578 (PMC13355637; doi:10.1016/j.jped.2026.101578)

**JPED-D-26-00024**

**Supplementary Material**


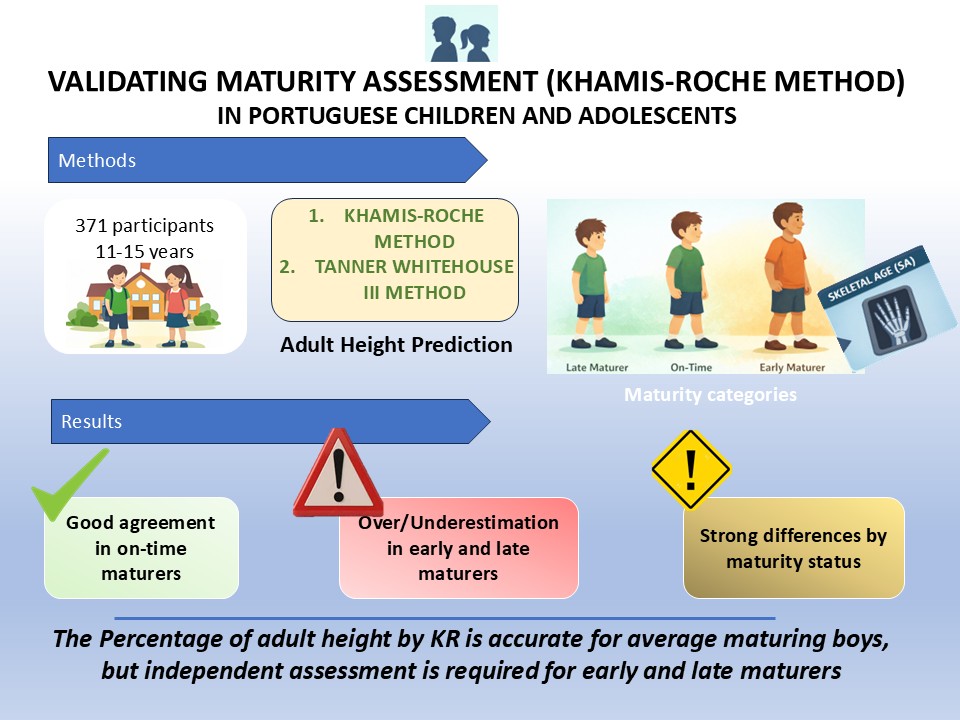

Supplement: Supplementary file 1 [file mmc1.docx]
